# Lip and Jaw Tremor in Parkinson’s Disease

**DOI:** 10.5334/tohm.1001

**Published:** 2025-04-14

**Authors:** José Fidel Baizabal-Carvallo, Marlene Alonso-Juarez, Robert Fekete

**Affiliations:** 1 Department of Sciences and Engineering, University of Guanajuato, León, México; 2 Instituto Politécnico Nacional, Mexico; 3 New York Medical College, Valhalla, New York, USA

**Keywords:** Parkinson’s disease, tremor, lip, jaw, clinical correlations

## Abstract

**Background:**

Parkinson’s disease (PD) is characterized clinically by the presence of bradykinesia, rigidity, and tremor. Although upper limb rest tremor is the most common form of tremor in PD, lip/jaw tremor is identified in a proportion of these patients.

**Methods:**

We aimed to assess the frequency, features, and correlates of lip/jaw tremor in PD.

**Results:**

We studied 229 consecutive patients with PD. There were 39 (17%) patients with lip/jaw tremor, 22 of them (56.4%) were males. Slight lip/jaw tremor was identified in n = 10 (25.6%), mild in n = 15 (38.5%), moderate in n = 13 (33.3%) and severe in n = 1 (2.6%) case. Patients with lip/jaw tremor had a positive association with older age, greater limb rest tremor scores (*P* = 0.009), and higher total MDS-UPDRS-III scores (*P* < 0.001) in the multivariate regression analysis. There were 6 patients with isolated lip/jaw tremor (i.e. without limb rest tremor). These patients were all male (*P* = 0.038), tended to be older (75.7 vs. 67.7 years, *P* = 0.078) and had greater cognitive impairment (*P* = 0.034) than the rest of the cohort, but there was no association with other body tremors or total motor score.

**Conclusions:**

Lip/jaw tremor was identified in 17% of cases; it was associated with greater motor severity and limb rest tremor, suggesting shared pathophysiology with limb rest tremor. A subgroup with isolated lip/jaw tremor showed reduced cognitive performance, but no association with other body tremors.

## Introduction

Parkinson’s disease (PD) is one of the most common neurodegenerative diseases, chiefly characterized by bradykinesia, rigidity, and tremor [1]. The latter is typically observed in the upper limbs, but it can also affect the lower limbs and the axial musculature. Rest tremor is reported to occur in between 65.4% and 69.6% of patients with early PD with an additional 9% of patients reporting tremor during follow-up [2,3]; whereas postural and kinetic tremor was observed in 50% of cases in a cohort of 378 patients with PD [3]. Jaw and lip tremors frequently coexist, and they probably represent the most common axial tremors in PD, secondary to oscillatory contractions of masticatory and perioral muscles. Jaw tremor commonly leads to the suspicion of underlying PD in individuals presenting for tremor evaluation [4]; moreover, it has been reported that clinical improvement of facial tremors following a levodopa challenge provides a 92% sensitivity and 93% specificity for the diagnosis of PD [5]. Despite this evidence, the frequency, clinical characteristics and correlations of lip and jaw tremor in patients with PD have been rarely studied. The characterizations of those features will help to better understand the pathophysiology of lip and jaw tremor to better assess whether these patients represent a particular subgroup in PD. For that reason, we aimed to evaluate these features in a cohort of patients with PD.

## Materials and Methods

We made a retrospective review of patients with PD attending a tertiary-care center for movement disorders. Patients were actively examined and asked for lip and jaw tremor during the first clinical encounter and follow-up visits. Patients were enrolled between August 2019 and November 2024. Inclusion criteria included patients of any age and both sexes diagnosed with PD according to the Queen Square Brain Bank (QSBB) criteria [6]. Exclusion criteria were patients with parkinsonism but with another disorder explaining such syndrome or without a clear diagnosis of PD. We excluded 8 patients with essential tremor (ET) and parkinsonism. However, patients with a clear diagnosis of PD according to the QSBB criteria and features suggesting underlying ET were included, such features comprised voice, head, or prominent kinetic arm tremor.

Demographic variables were recorded in each case, including sex and age at evaluation and time since onset of motor symptoms. The neurological evaluation of all patients was performed by a movement disorders specialist (JFB-C) with the Movement Disorders Society Unified Parkinson’s Disease Rating Scale part III or motor score (MDS-UPDRS-III) [7]. Limb bradykinesia was summarized in a composite score by adding items (3.4, 3.5 and 3.6) of both arms and items (3.7 and 3.8) of both legs (range: 0–40); axial and limb rigidity was summarized in a composite score by adding the individual scores of the neck and four limbs from item 3.3 (range: 0–20). Upper limb postural (item 3.15) and kinetic tremors (item 3.16) were expressed in composite scores by adding the scores on each side (range: 0–8, for each); whereas limb rest tremor was summarized using item 3.17 by adding the rest tremor score of the four limbs (range: 0–16). Lip/jaw tremor (item 3.17) and tremor persistence (item 3.18) were assessed separately. The amplitude of lip/jaw tremor was estimated according to item 3.17. as 0) no tremor, 1) slight: <1 cm maximal amplitude, 2) mild: ≥1 to <2 cm of maximal amplitude, 3) moderate: ≥2 cm but <3 cm in maximal amplitude, and 4) severe: ≥3 cm in maximal amplitude [7].

Patients were evaluated in the clinical off state between 6 and 12 hours after the last dose of levodopa, whereas n = 90 (39.3%) patients were levodopa naïve at baseline examination. The disease stage was assessed with the modified Hoehn & Yahr (HY) scale [8]. The cognitive evaluation was carried out with the Montreal Cognitive Assessment (MoCA). The antiparkinsonian medications taking at the time of evaluation were registered for each patient and the levodopa equivalent daily dose (LEDD) was calculated for each case [9]. The study was approved by our local Internal Review Board.

## Statistics

Data were summarized in percentages, means with standard deviations. The X^2^ test with Yates’s continuity correction or the Fisher*’*s exact test when it applies, were used to compare nominal or ordinal data between groups. We carried out a multivariate logistic regression analysis with the backward Wald*’*s method and “presence of lip/jaw tremor” as the dependent variable to assess the association of variables showing a statistically significant result in the bivariate analysis. The (exp)^B^ coefficient as odds ratios with 95% confidence interval (C.I.) were used to standardize the weight of independent variables and assess their association with the presence of lip/jaw tremor. The Hosmer-Lemeshow test was used to assess goodness of fit and the Nagelkerke test to calculate the determination R^2^ coefficient of the regression model. All statistical calculations were performed using SPSS version 22, a *P* value < 0.05 was considered significant.

## Results

There were 229 consecutive patients with PD included in the study, n = 130 (56.8%) were males and n = 99 (43.2%) were females. The mean age at evaluation was 67.78 ± 11.09 years and 6.60 ± 6.60 years since onset of motor symptoms for the whole cohort. A total of 39 (17%) patients were identified with lip/jaw tremor. No patient endorsed lip/jaw tremor as the first symptom of PD, as this tremor appeared during the evolution of the disease. According to item 3.17, the severity of lip/jaw tremor was n = 10 (25.6%) for slight, n = 15 (38.5%) for mild, n = 13 (33.3%) for moderate and n = 1 (2.6%) for severe. The frequency of lip/jaw tremor did not differ between patients who were levodopa naïve and those taking levodopa, 16.6% vs. 17.3% respectively, (*P* = 0.906). All patients had rest lip/jaw tremor, moreover, a postural component was observed in 24 patients, whereas 5 patients had kinetic jaw tremor (i.e., appearing while speaking). Isolated jaw tremor (i.e., without accompanied rest tremor in the limbs or other axial muscles) was observed in 6 (2.6%) cases. Lip/jaw tremor was observed to improve with adjustment of dopaminergic therapy in 33 cases, 4 patients did not show improvement and 2 patients were lost at follow-up. There was an improvement in the mean item 3.17 score from 2.14 at baseline to 0.84 at follow-up (between 3 and 6 months) after adjustment of dopaminergic therapy (*P* < 0.001).

When contrasting the clinical and demographic features, those patients with lip/jaw tremor were older (*P* = 0.065) and had higher composite scores in rigidity (*P* = 0.002), bradykinesia (*P* = 0.001), postural tremor (*P* < 0.001), kinetic tremor (*P* = 0.010), rest tremor (*P* < 0.001) and tremor persistence (*P* < 0.001). Some axial features showed also higher scores in patients with jaw tremor, including facial expression (*P* = 0.036), postural stability (*P* = 0.002) and posture (*P* = 0.018). The total MDS-UPDRS-III score (*P* < 0.001) and modified HY scale (*P* = 0.005) were also higher in patients with lip/jaw tremor (Table 1). No differences were observed for sex distribution or evolution time, between patients with and without lip/jaw tremor.

**Table 1:** Contrasting features between Parkinson’s disease patients with and without jaw tremor.


	JAW TREMOR PRESENCE n = 39 (%)	JAW TREMOR ABSENCE n = 190 (%)	*P*-VALUE

Sex (male)	22 (56.4)	108 (56.8)	0.960

Age (years)	70.77 ± 11.22	67.17 ± 10.99	0.065

Evolution time (years)	8.19 ± 8.43	6.27 ± 6.14	0.100

Cognitive status (MoCA)	21.06 ± 5.36	21.69 ± 5.83	0.668

**MDS-UPDRS-III (subscores)**			

Language	0.92 ± 1.09	0.96 ± 1.05	0.829

Facial expression	1.44 ± 1.02	1.09 ± 0.92	**0.036**

Rigidity composite score	9.33 ± 4.86	7.05 ± 4.03	**0.002**

Bradykinesia composite score	18.38 ± 9.77	13.02 ± 9.09	**0.001**

Rising from chair (3.9)	1.51 ± 1.62	1.18 ± 2.39	0.406

Gait (3.10)	2.08 ± 1.27	1.69 ± 1.16	0.061

Freezing (3.11)	0.79 ± 1.51	0.66 ± 1.24	0.540

Postural stability (3.12)	2.15 ± 1.25	1.50 ± 1.17	**0.002**

Posture (3.13)	1.59 ± 1.14	1.17 ± 0.95	**0.018**

Postural tremor (composite score)	2.08 ± 1.86	1.06 ± 1.33	**<0.001**

Kinetic tremor (composite score)	1.56 ± 1.68	0.97 ± 1.22	**0.010**

Rest tremor (composite score)	4.97 ± 3.46	1.85 ± 2.31	**<0.001**

Tremor persistence	3.03 ± 0.873	1.25 ± 1.38	**<0.001**

Total score	52.56 ± 22.89	34.53 ± 17.52	**<0.001**

**Hoehn-Yahr scale** (modified)	3.62 ± 0.96	3.17 ± 0.88	**0.005**

Dyskinesia presence, n (%)	5 (12.8)	28 (14.7)	0.952 cc

**Treatment**			

Levodopa/carbidopa, n (%)	24 (61.5)	115 (60.5)	0.966

Amantadine, n (%)	4 (10.3)	13 (6.8%)	0.502

Dopamine agonists, n (%)	10 (25.6)	52 (27.4)	0.981

Anticholinergic, n (%)	5 (12.8)	11 (5.8)	0.159

MAOI, n (%)	2 (5.1)	20 (10.5)	0.385

**LEDD**	458.3 ± 425.5	595.1 ± 632.6	0.101


LEDD: Levodopa equivalent daily dose; MAOI: Monoamine oxidase inhibitor.

In the multivariate analysis, the following variables: age, rest tremor, and total MDS-UPDRS-III score were retained in the final regression model with a positive association with jaw tremor; other independent variables such as the modified HY scale, composite scores in bradykinesia, rigidity, postural and kinetic tremor, or subscores in facial expression, postural stability, and posture were not included in the final regression model (Table 2).

**Table 2:** Multivariate analysis of statistically significant variables in the bivariate analysis.


VARIABLES IN THE FINAL REGRESSION MODEL	EXP^B^ COEFFICIENT	EXP^B^ 95% C.I.	*P*-VALUE

Age	1.035	0.996–1.076	**0.075**

MDS-UPDRS-III score	1.029	1.007–1.051	**0.009**

Rest tremor composite score	1.383	1.197–1.597	**<0.001**


Constant: B-6.220, Exp^B^: 0.00, *P* < 0.001. Hosmer-Lemeshow: (*P* = 0.299). Nagelkerke R^2^ = 0.323. Variables not included in the final equation: Hoehn-Yahr scale, postural tremor composite score, kinetic tremor composite score, facial expression, bradykinesia composite score, rigidity composite score, postural stability and posture, scores. MDS-UPDRS-III: Movement Disorders Society Unified Parkinson’s Disease Rating Scale, part III.

There were 6 patients with isolated lip/jaw tremor, they were all male (*P* = 0.038), tended to be older (75.7 vs. 67.7 years, *P* = 0.078), had a poorer cognitive performance (MoCA scores: 14.7 vs. 21.8, *P* = 0.034) and worse postural stability scores (2.67 vs. 1.58, *P* = 0.029) than the rest of the cohort. However, no statistically significant differences were observed in HY scale, evolution time, sub- and total motor scores (MDS-UPDRS-III) or LEDD (*P* > 0.05, for all those comparisons).

## Discussion

In this study, we identified jaw tremor in 17% of patients with PD. This frequency is like the 18% prevalence reported in a large cohort (n = 2224) of Chinese patients with PD [10]. However, it is possible that we underestimated the frequency of lip/jaw tremor, as some patients may not report this type of tremor as it may not be present during the clinical examination.

In the study by Ou and colleagues, patients with facial tremor (involving lip and jaw tremor) had longer evolution times and were more commonly females [10]; this was not observed in our study, moreover, other cohort studies have shown that the prevalence of rest tremor in PD tends to be stable at follow-up and its frequency does not seem to differ between males and females [2,3].

The bivariate analysis in our study showed that certain motor features were more severe in patients with lip/jaw tremor, including facial expression, rigidity, bradykinesia, posture, and postural stability, as well as postural, kinetic and rest tremor. In the Chinese study, jaw tremor was associated with axial features [10]. However, the multivariate analysis showed a statistically significant positive association between lip/jaw tremor and the total motor score and the composite rest tremor score that included the upper and lower limbs. The strong association between lip/jaw tremor and rest tremor of the limbs, suggests that both types of tremors may have a similar underlying pathophysiology but different somatotopy. The interactions between high power oscillations in the basal ganglia and cerebello-thalamic-cortical circuits have been proposed in the “dimmer-switch model” of PD tremor. This model predicts a tremor trigger most likely in the basal ganglia and a tremor enhancer of amplitude in the cerebello-thalamic-cortical projections [11,12].

We identified a small group of patients with isolated lip/jaw tremor (i.e. without rest limb tremor). These patients were older and showed greater cognitive impairment and worse postural stability than the rest of the cohort, but there was no association with other motor scores. The nature of this finding is unclear; however, it is worth noting that patients with PD and dementia had greater frequency of axial manifestations, which may possibly also involve axial tremor in selected cases [13].

Lip/jaw tremor in PD is usually at rest, observed with closed mouth, but it may also be observed with some degree of mouth opening, suggesting the presence of postural tremor, this was observed in a proportion of patients in our study. However, while kinetic jaw tremor, for example while drinking water or speaking, raises the suspicion of underlying ET, improvement with levodopa therapy supports the notion that the underlying cause is rather PD [14].

Lip/jaw tremor should be differentiated from other tremor syndromes affecting the cranial muscles (Table 3). In this regard, ET is the most common differential diagnosis. Jaw tremor in ET has been reported in 7.5% of individuals in a population-based study; in 10% in a tertiary referral and in 18% in a brain repository sample [14,15]. Other conditions causing oscillatory movements in the central facial-cranial musculature resembling tremor such as jaw clonus, hereditary geniospasm, rabbit syndrome, myorhythmia or rhythmic stereotypies are classical descriptions that should also be differentiated from jaw tremor observed in PD (Table 3) [16,17,18,19,20,21]. However, other disorders such as dystonic jaw tremor, branchial myoclonus, and functional jaw movements should also be differentiated from jaw tremor in PD. The latter usually shows the clinical features of other functional movement disorders, such as distractibility, suggestibility, sudden onset, episodes of unexplained improvement or worsening, besides other incongruencies in clinical phenomenology with “non-functional” or “organic” movement disorders [22].

**Table 3:** Contrasting clinical features of rhythmic repetitive lip/jaw movements.


	PARKINSON’S JAW TREMOR	ESSENTIAL JAW TREMOR	JAW CLONUS	GENIOSPASM	RABBIT SYNDROME	TASK-SPECIFIC JAW TREMOR	MYORHYTHMIA	TARDIVE PHENOMENA

**Etiology**	PD and other parkinsonian syndromes	Essential tremor	ALS, NMO, OD	Mostly hereditary, isolated in few cases	Drug-induced by DRB	Unknow	Stroke, infections (WpD), autoimmune	Tardive dyskinesia or tremor (DRB and other drugs)

**Prevalence within specific cause**	17–18%	7.5 to 18%	Rare	All cases by definition	2.3 to 4.4%	Rare	Rare	Common (dyskinesia), tremor (uncommon)

**Type of movement**	Jaw and lip rest tremor, postural and kinetic component are less common	Jaw tremor is usually postural or kinetic	Elicited by testing the jaw-jerk reflex or by jaw opening	Rhythmic or irregular twitch-like chin quivering or trembling in bouts lasting minutes to hours	Vertical mouth and lip movements, without tongue involvement	Jaw tremor triggered with a specific task (i.e., drinking water)	Continuous central facial rhythmic movements, + nystagmus, palatal, limb movements (OMM)	Repetitive masticatory movements or jaw tremor + lingual, limb or axial stereotypies

**Movement frequency (Hz)**	3–5 Hz	4–12 Hz	~ 10 Hz	5–6 Hz	~ 5 Hz	5–8 Hz	1–4 Hz	1–2 Hz

**Clinical correlates and/or comorbid conditions**	Older age, greater limb rest tremor severity, higher motor scores	Older age, presence of head and voice tremor, greater arm tremor severity, rest tremor in the arms.	Upper motor lesion above the spinal cord	Family history (Autosomal dominant pattern). RNTB	Underlying schizophrenia, bipolar disorder, Korsakoff’s sx, etc. Some have parkinsonism	Some may improve with alcohol, or alleviating maneuvers (i.e., sensory tricks)	SnGP, arthralgia, diarrhea, weight loss or focal deficits depending on the cause	Associated psychiatric disorders. Some have parkinsonism

**Treatment**	Levodopa, dopamine agonists, Achol, BoNT	Propranolol, primidone, topiramate, BoNT	Baclofen, tizanidine, BZD, BoNT	BoNT; BZD for RNTB	↓ DRB or withdrawal, Achol	BoNT	Treat underlying cause, i.e., Abs for WpD	↓ DRB or withdrawal, TBZ, VBZ, deuTBZ


Abs: antibiotics; Achol: anticholinergics (benzatropine, procyclidine, trihexyphenidyl, etc.); ALS: amyotrophic lateral sclerosis; BoNT: botulinum toxin; BZD: benzodiazepines; deuTBZ: deutetrabenazine; DRB: Dopamine receptor blockers; NMO: neuromyelitis optica; OD: osmotic demyelination; OMM: oculomasticatory myorhythmia; PD: Parkinson’s disease; RNTB: Recurrent nocturnal tongue biting; SnGP: supranuclear gaze palsy; Sx: syndrome; TBZ: tetrabenazine; VBZ: valbenazine; WpD. Whipple disease.

Improvement of jaw tremor with dopaminergic medication was observed in most patients in our cohort, after adjusting dopaminergic therapy. While jaw tremor in PD usually improves with levodopa, dopaminergic agonists, anticholinergics, and amantadine; botulinum toxin (BoNT) may be an alternative for patients with high amplitude jaw tremor, as such severe jaw oscillations may lead to temporo-mandibular joint dysfunction, continuous teeth friction, repetitive annoying noises, or tongue biting [23]. Moreover, other complications of rest tremor in oropharyngeal structures were reported in 34 patients with PD, as these patients had more commonly diverse combinations of altered timing of swallow events, increased pharyngeal residue and greater events of airway invasion by thinner and larger volume boluses [24]. Further studies should clarify how lip/jaw tremor may interfere with swallowing or airway aspiration.

Our study has limitations, one is that the prevalence of lip/jaw tremor may be underestimated, as tremor may be an intermittent phenomenon not observed during the clinical encounter and subject to recall bias by patients. Another limitation is that we did not carry out neurophysiology tests to assess the time-frequency, amplitude, and coherence of jaw tremor with other body tremors. Tremor in PD has been subdivided in 4 phenomenological categories: type I, pure resting (4–6 Hz) tremor; type II, rest tremor plus action tremor with same frequency (Hz); type III, isolated action tremor and type IV rest tremor plus action tremor with different frequencies (Hz) [25]. However, it is unclear whether this classification can be used for lip/jaw tremor as subcategories, and this topic should be further explored with neurophysiology studies. We did not assess specifically for motor fluctuations; however, it is possible that during the wearing off phenomenon, patients with PD may display lip/jaw tremor that may be visible during the clinical examination. While not all patients were strictly in the 12-hour off state, the frequency of lip/jaw tremor did not differ between patients’ levodopa naïve and patients taking levodopa (*P* = 0.906).

## Conclusions

Lip/jaw tremor is identified in 17% of patients with PD, it is usually mild to moderate in amplitude, presents at rest, but it may have a postural or kinetic component in a proportion of cases with improvement typically following adjustment of dopaminergic therapy. It associates with greater severity of limb rest tremor, suggesting a similar underlying pathophysiology. A small proportion of these patients presented with isolated lip/jaw tremor, which associated with older age and greater cognitive impairment.

## Data Accessibility Statement

Data is available on request to the authors.

## Abbreviations

BoNT: Botulinum toxin

ET: essential tremor

HY: Hoehn-Yahr scale

MDS-UPDRS-III: Movement Disorders Society Unified Parkinson’s Disease Rating Scale part III

MoCA: Montreal Cognitive Assessment

PD: Parkinson’s disease

QSBB: Queen Square Brain Bank
